# Differences in Tsimane children’s growth outcomes and associated determinants as estimated by WHO standards vs. within-population references

**DOI:** 10.1371/journal.pone.0214965

**Published:** 2019-04-17

**Authors:** Melanie Martin, Aaron Blackwell, Hillard Kaplan, Michael Gurven

**Affiliations:** 1 Department of Anthropology, University of California Santa Barbara, Santa Barbara, CA, United States of America; 2 Department of Anthropology, University of New Mexico, Albuquerque, NM, United States of America; Baylor University, UNITED STATES

## Abstract

Anthropometric measures are commonly converted to age stratified z-scores to examine variation in growth outcomes in mixed-age and sex samples. For many study populations, z-scores will differ if calculated from World Health Organization (WHO) growth standards or within-population references. The specific growth reference used may influence statistical estimates of growth outcomes and their determinants, with implications for biological inference. We examined factors associated with growth outcomes in a sample of 152 Tsimane children aged 0–36 months. The Tsimane are a subsistence-scale population in the Bolivian Amazon with high rates of infectious disease and growth faltering. To examine the influence of growth reference on statistical inferences, we constructed multiple plausible models from available infant, maternal, and household attributes. We then ran identical models for height-for-age (HAZ), weight-for-age (WAZ), and weight-for-height (WHZ), with z-scores alternately calculated from WHO and robust Tsimane Lambda-Mu-Sigma growth curves. The distribution of WHO relative to Tsimane HAZ scores was negatively skewed, reflecting age-related increases in lower HAZ. Standardized coefficients and significance levels generally agreed across WHO and Tsimane models, although the strength and significance of specific terms varied in some models. Age was strongly, negatively associated with HAZ and WAZ in nearly all WHO, but not Tsimane models, resulting in consistently higher R^2^ estimates. Age and weaning effects were confounded in WHO models. Biased estimates of determinants associated with WHO HAZ may be more extreme in small samples and for variables that are strongly age-patterned. Additional methodological considerations may be warranted when applying WHO standards to within-population studies, particularly for populations with growth patterns known to systematically deviate from those of the WHO reference sample.

## Introduction

Anthropometric measures of body size are widely used to assess growth, nutritional status, and biological fitness [1–3]. For mixed age and sex samples, these measures are often converted to age-stratified z-scores (i.e. height-for-age, HAZ; weight-for-age, WAZ; weight-for-height z-score, WHZ), calculated against a large internal or established external reference—e.g. the U.S. CDC growth charts or the World Health Organization (WHO) Growth Standards [4]. The current WHO standards, in place since 2006, were derived from a large, longitudinal multi-ethnic survey and are ideal for cross-population comparisons. Unlike the previous CDC/NCHS reference, the WHO standards also importantly established growth of breastfed infants—who have slower growth trajectories than formula-fed infants—as the normative baseline for children 0–24 months of age [5–7].

However, the distinction between “reference” and “standard” has meaningful methodological implications. A “reference” represents growth outcomes in a particular place and time, whereas the WHO “standard” represents optimal growth potential, i.e. how children “*ought to grow under optimal conditions”* [5,8,9]. Systemic negative deviations from the WHO standards are generally interpreted as evidence of suboptimal growth attributed to nutritional and pathogenic exposures. However, genetic and other factors also influence population-specific growth trajectories [10–14]. For example, the WHO standards have been shown to alternately under- or overestimate the prevalence of stunting, underweight, and overweight in affluent populations in China, Japan, and India [15–18]. Even populations included in the WHO reference samples are not fully represented, as the survey excluded low socioeconomic status families, families living above 1500 m altitude, mothers who smoked during pregnancy or lactation, children born at < 37 weeks or ≥ 42 weeks, and children with substantial morbidities [5,14]. As such, an individual child’s growth relative to other children in their population will always be more faithfully represented by within-population z-scores, even within affluent populations [4,19].

Cole advises considering whether the goal is to examine variation in “healthy growth” or “representative growth” in deciding whether to use the standards or a local reference, if available [8]. Kramer et al. have further cautioned that deviance from an optimal standard may have little bearing on a child’s relative biological fitness within a population [20]. We further propose that the choice of local reference vs. growth standard may differently influence statistical relationships between estimated growth outcomes and locally varying social, economic, or biological factors—with implications for inferring biological relevance. Mean WHO HAZ scores decline systematically across early childhood in resource poor-settings due to nutritional and infectious conditions [21–24], resulting in increased age-related variance in WHO HAZ scores that may bias parameter estimates in mixed-age samples [25,26]. Although researchers often control for child age in statistical models [27,28,29], the systematic deviance in WHO-derived z-scores may bias or confound estimates of size differences associated with locally varying determinants, particularly those correlated with age or developmental changes. In contrast, within-population growth references should minimize the influence of endemic influences in estimating relative size, resulting in more accurate and biologically relevant estimates of local growth determinants in regression models.

The Tsimane are a high-fertility, high-mortality population of forager-horticulturalists residing in the Bolivian Amazon [30]. Tsimane infants are exclusively breastfed for four months and weaned later than two years on average [31]. However, no Tsimane households have access to improved or safely managed water sources, and few households have electricity. Endemic parasitism and infectious diseases impose substantial immune and energetic costs [31–34]. Infant mortality rates have been previously estimated at more than double the national rates for Bolivia—largely owing to respiratory and gastrointestinal infection [35]—with higher parity and shorter IBI associated with increased risk of infant mortality and growth faltering [36,37]. In previous surveys of child nutritional status as assessed by WHO standards, 47% of children aged 0–5 were classified as stunted and 18% as underweight [12]. Given the co-occurrence of protective and risk factors that may influence Tsimane growth patterns (e.g. prolonged breastfeeding vs. endemic infectious disease), developing appropriate initiatives to improve Tsimane child welfare requires accurate identification of local growth determinants and at-risk individuals.

We examined growth outcomes of Tsimane children aged 0–36 months in association with different age-related and fixed infant, maternal, and household variables. We assessed model parameters in side-by-side comparisons of identical models with HAZ, WAZ, and WHZ scores calculated from WHO and Tsimane Lambda-Mu-Sigma (LMS) growth curves. The Tsimane LMS curves were generated from 30,118 mixed-longitudinal measures from 9,614 individuals, using methods identical to those used in formulating WHO standards, allowing for robust comparisons [12,38]. We observed that coefficient estimates and significance levels generally agreed between WHO and Tsimane-derived WAZ and WHZ models, but differed for specific terms in HAZ models, largely owing to age-related confounding in WHO scores.

## Materials and methods

### Data collection

MM conducted a mixed-longitudinal study of infant feeding practices and maternal and infant health outcomes across nine Tsimane villages from September 2012—April 2013. The villages varied with respect to river access and distance to the market town of San Borja (pop. ~24,000). All families with children aged 0–35 months were asked to participate, resulting in a sample of 156 families from 150 households, and representing 92% of all eligible families present. Anthropometric measures were collected in participants’ homes during initial ethnographic interviews. A subsample of 41 infants who were less than one year of age at the time of initial interview were recruited for prospective follow-up study, with follow-up measures taken approximately every 6 weeks for the next 8 months. Subjects followed prospectively contributed 2–6 measures total (mean ± SD = 3.2 ± 1.4 measures per subject), with the number of measures varying due to age at entry and intermittent absences. A total of 287 anthropometric measures are included in the final mixed-longitudinal sample (156 from initial interviews, 131 from follow-up). Male infants and remote villages were over-represented in the follow-up group as compared to the cross-sectional only group (Table 1).

**Table 1:** Sample characteristics.

	All Participants	Prospective only (initial observations)	Cross-sectional only
Characteristic	n = 156	n = 41	n = 115
	Mean ± SD (Range) n (%)	Mean ± SD (Range) n (%)	Mean ± SD (Range) n (%)
Maternal age (yrs)	27.3 ± 8.5 (14.1–49.7)	27.9 ± 9.0 (14.1–45.3)	27.0 ± 8.3 (14.5–49.7)
Mat. height (cm)	152.0 ± 4.6(141.7–170.1)	151.4 ± 5.3 (143.4–170.1)	152.2 ± 4.3 (141.7–162.7)
Mat. parity	4.5 ± 2.9 (1–13)	5.0 ± 3.1 (1–12)	4.3 ± 2.9 (1–13)
Primiparous	27 (17.3%)	6 (14.6%)	21 (18.3%)
2–6 births	94 (60.3%)	24 (58.5%)	70 (60.9%)
≥ 7 births	35 (22.4%)	11 (26.8%)	24 (20.9%)
IBI*	33.0 ± 21.4 (10.7–164.6)	37.6 ± 25.2 (14.3–160.6)	31.3 ± 19.7 (10.7–164.6)
First born	27 (17.5%)	6 (14.6%)	21 (18.6%)
< 33 months	87 (56.5%)	22 (53.7%)	65 (57.5%)
≥ 33 months	40 (25.6%)	13 (31.7%)	27 (23.9%)
EBF duration *	3.8 ± 2.0 (0–7)	3.2 ± 1.9 (0–6)	3.9 ± 2.1 (0–7)
0–3 months*	54/129 (41.9%)	9 (37.5%)	39 (37.1%)
≥ 4 months*	75/129 (58.1%)	15 (62.5%)	66 (62.9%)
Breastfeeding status			
EBF	26/43 (60.5%)	16/26 (61.5%)	10/17 (41.2%)
Weaned	23/113 (20.4%)	0/15 (0%)	23/98 (23.5%)
Village region			
Near market	87 (55.8%)	10 (24.4%)	77 (67.0%)
Remote	69 (44.2%)	31 (75.6%)	38 (33.0%)
Sex			
Male	89 (57.1%)	27 (65.8%)	62 (53.9%)
Female	67 (42.9%)	14 (34.1%)	53(46.1%)
Birth season			
Dry	76 (48.7%)	20 (48.8%)	56 (48.7%)
Rainy	80 (51.3%)	21(51.2%)	59 (51.3%)

Descriptive statistics of child participants and households at first interview are further grouped according to cross-sectional and prospective follow-up samples.

* Exclusive breastfeeding duration (EBF) reported for non-EBF children only (n = 129). Breastfeeding status reported separately for children 0–5 months (n = 43) and 6–35 months (n = 113).

Infant recumbent length was measured to the nearest 0.5 cm using a pediatric measure mat. Maternal and child standing heights were measured to the nearest 0.1 cm using a portable Seca 217 stadiometer. All heights were measured in duplicate and averaged in the event of a discrepancy. Maternal and child weights were measured to the nearest 0.1 kg with a digital scale (Tanita BF680W Duo Scale), using the tare method to weigh infants-in-arms. The scale was placed on a small raised wooden platform to minimize measurement error on the uneven surfaces of participant homes. All subjects were weighed fully clothed (Tsimane women typically wear lightweight skirts and tops). Infants and young children are typically dressed in only a t-shirt or a t-shirt with lightweight cotton pants (they do not wear diapers). Infants were removed from swaddling materials before measurement. Weight and standing height were measured barefoot.

Current feeding status (exclusive breastfeeding, breastfeeding, weaned) was determined by maternal 24-hour recall reported at all interviews. Age of complementary feeding (CF) introduction was recorded from maternal recall at initial interview (for non-exclusively breastfeeding children) or when a change in feeding status was first reported (for exclusively breastfeeding children in the prospective sample). Birth order, preceding interbirth interval, and number of live siblings under the age of five were determined through maternal interviews and checked against family health records and demographic and census information previously collected by the Tsimane Health and Life History Project [30].

### Ethics statement

All study protocols were approved by the University of California Santa Barbara Institutional Review Board. Participant consent and approvals to conduct the research in Bolivia were obtained through several channels. The Tsimane Health and Life History Project maintains formal agreements with the local municipal government of San Borja and the Tsimane governing body (*Gran Consejo Tsimane’*) to conduct research with Tsimane communities. MM additionally arranged independent agreements to conduct the present study with the Gran Consejo and leaders of participating study communities. In compliance with national requirements for conducting scientific research in Protected Areas of Bolivia, approval for the study was granted from the Estación Biológica del Beni and the Ministerio del Medio Ambiente y Agua.

The purpose of the study was explained to each of the study villages in community meetings held prior to beginning data collection and individually during participant recruitment. Participants gave verbal informed consent before each interview and follow-up visit, as most Tsimane women are illiterate. Mothers gave verbal consent for infant participation. Verbal consent was not recorded. The verbal consent procedure was described in the protocol approved by the Institutional Review Board. Participants were compensated with small care packages that included household goods (e.g. yarn, thread, combs) and over-the-counter medicines (e.g. paracetamol, salve).

### Z score calculations and statistical analyses

WHO and Tsimane LMS HAZ, WAZ, and WHZ scores were calculated using the open-source ‘*localgrowth’* R package (https://github.com/adblackwell/localgrowth), which was developed from previously published databases and R code [12,38]. To maintain comparable sample sizes across models, we removed observations from two subjects with unknown previous IBIs, and four observations with missing height or weight measures. The final sample included 152 subjects and 281 mixed-longitudinal measures.

We examined WHO- and Tsimane-derived z-scores in association with several locally relevant static and age-related independent variables expected to influence growth outcomes. Factors expected to be associated with poorer growth outcomes included shorter interbirth intervals and higher parity [39–41], remote village residence or rainy season birth [42–45] greater number of household dependents under the age of five [46], and relatively early complementary feeding introduction (0–3 months) or weaning [47,48] (see S1 Text for extended discussion of selected variables). We first constructed a baseline linear mixed-effects model consisting of child ID as a random effect, and child sex, age (in months), and maternal height as fixed effects. This baseline model was run separately for each of WHO and Tsimane HAZ, WAZ, and WHZ scores, using the full sample of all observations for children aged 0–35 months (n subjects = 152, n observations = 28, see Models 1a-e in S1 Table). The following variables were then considered additively in separate models (Models 2-6a-e in S1 Table): previous IBI, birth order, number of dependents under the age of five, village distance to market, and birth season. Additional models considered age-related feeding practices—duration of exclusive breastfeeding and weaning status—and were run on age-specific subsets: exclusively breastfeeding vs. breastfeeding with complementary feeding (ages 0–5 months, Models 7a-f in S1 Table); complementary feeding introduction at 0–3 vs. 4–6 months (ages 6–35 months, Models 8a-f in S1 Table); breastfeeding vs. weaned (ages 6–35 months, Models 9a-f in S1 Table). Because the aim of this study was to compare statistical relationships between independent variables and WHO- vs. Tsimane-derived z-scores in a variety of plausible models, we did not correct for multiple comparisons or apply model selection criteria to individual models.

All models were run using the *lme4* package in R. AIC and BIC were extracted from summary results. Due to differences in sample sizes, AIC values are only comparable for models 1a, 1b – 6a, 6b. Wald confidence intervals were extracted from standard errors using built-in commands. Marginal and conditional R^2^ values and p-values were estimated using command features of the *piecewiseSEM* and *lmerTest* packages. Data and R code are publicly available at https://figshare.com/projects/Shared_data_Tsimane_vs_WHO_comparisons/58187 (10.6084/m9.figshare.7496321 and 10.6084/m9.figshare.7496339)

## Results

### Descriptive statistics

**Fig 1:**
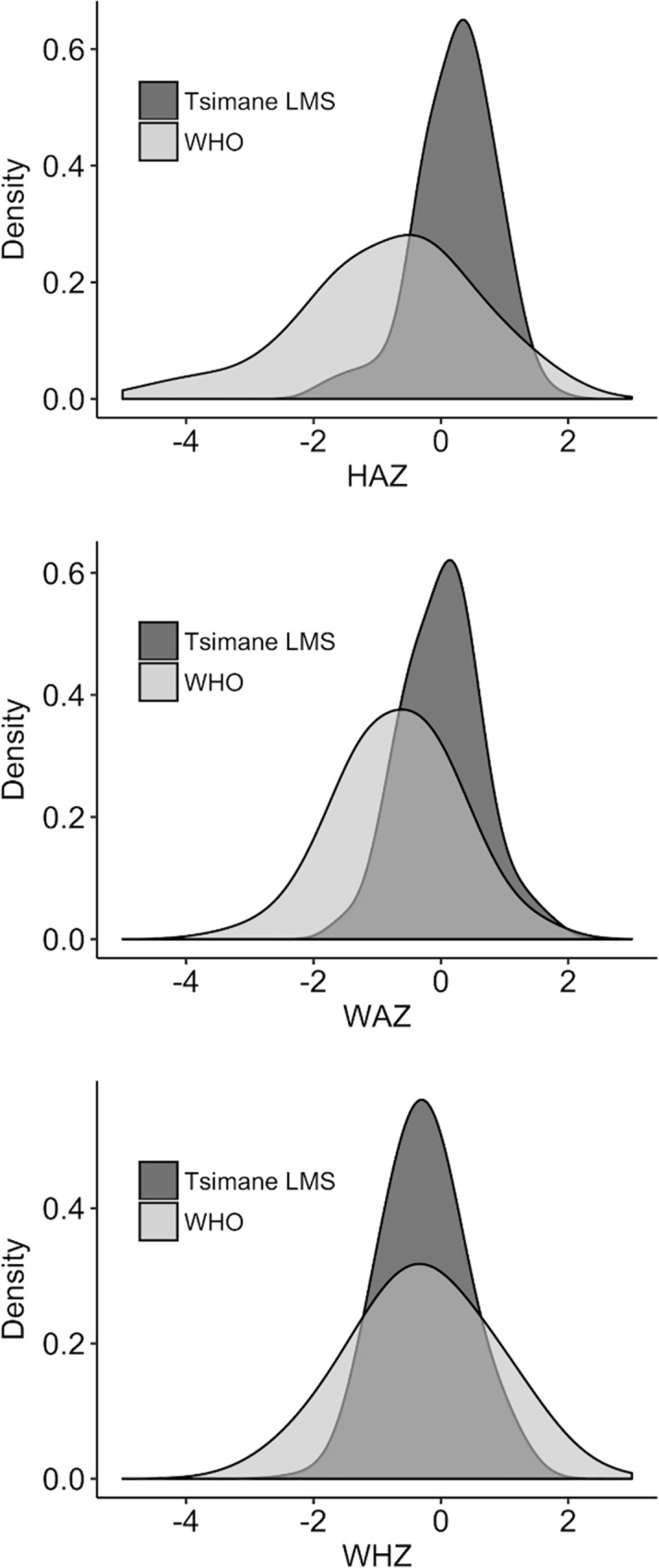
**A-C. Density plots of WHO- and Tsimane LMS-derived HAZ, WAZ, and WHZ scores.** Plots reflect measures collected from all subjects aged 0–35 months (n subjects = 156; n observations = 287). Fig 1A HAZ (top); Fig 1B WAZ (middle); Fig 1C WHZ (bottom).

Tsimane LMS and WHO-derived z-scores were highly correlated across all observations, most strongly for WHZ (HAZ *r =* 0.84, *p* < 0.001, WAZ *r* = 0.91, *p* < 0.001, WHZ = 0.94, < 0.001). However, the distributions of WHO- as compared to Tsimane-derived z-scores were more platykurtic. WHO-derived HAZ scores show a clear left skew, while WAZ and WHZ distributions were approximately more normal (Fig 1A–1C). Table 2 displays the prevalence of -2 and -3 standard deviations (SD) for WHO and Tsimane LMS z-scores at initial interviews for all age groups. For WHO-derived z scores only, age group was associated with variance in HAZ (F = 19.78, df = 3, 151, p < 0.001) and WAZ (F = 6.25, df = 3,151, p = 0.002), but not WHZ (F = 1.13, df = 3,150, *p* = 0.34).

**Table 2:** WHO and Tsimane LMS z-scores.

Age group (months)	N	Mean	SD	Range	% < -2SD	% < -3SD
**WHO** standards WAZ						
(0–5)	43	-0.26	0.86	(-1.78–1.90)	0 (0%)	0 (0%)
(6–11)	26	-0.44	1.05	(-2.61–1.68)	3 (11.5%)	0 (0%)
(12–23)	61	-0.98	0.96	(-3.72–1.24)	7 (11.5%)	3 (5%)
(24–35)	25	-1.10	0.97	(-2.99–0.80)	4 (16%)	0 (0%)
Total:	155	-0.71	1.01	(-3.72–1.90)	14 (9.0%)	3 (1.9%)
**WHO** standards HAZ						
(0–5)	43	-0.09	1.12	(-4.48–1.86)	0 (0%)	1 (2.3%)
(6–11)	27	-0.80	1.09	(-2.66–0.75)	4 (14.8%)	0 (0%)
(12–23)	60	-1.63	1.39	(-4.75–1.42)	12 (20%)	10 (16.7%)
(24–35)	25	-2.14	1.13	(-4.96–0.35)	7 (28%)	5 (20%)
Total:	155	-1.14	1.44	(-4.96–1.86)	23 (14.4%)	16 (10.2%)
**WHO** standards WHZ						
(0–5)	43	-0.25	1.11	(-2.54–2.75)	1 (2.3%)	0 (0%)
(6–11)	26	0.03	1.00	(-1.79–1.87)	0 (0%)	0 (0%)
(12–23)	60	-0.25	1.03	(-2.91–1.94)	3 (5.0%)	0 (0%)
(24–35)	25	0.12	1.00	(-2.39–1.53)	1 (4%)	0 (0%)
Total:	154	-0.14	1.05	(-2.91–2.75)	5 (3.2%)	0 (0%)
**Tsimane** reference WAZ						
(0–5)	43	-0.01	0.53	(-0.92–1.23)	0 (0%)	0 (0%)
(6–11)	26	0.20	0.69	(-1.05–1.81)	0 (0%)	0 (0%)
(12–23)	61	-0.03	0.68	(-1.62–1.76)	0 (0%)	0 (0%)
(24–35)	25	0.01	0.86	(-1.56–1.78)	0 (0%)	0 (0%)
Total:	155	0.02	0.68	(-1.62–1.81)	0 (0%)	0 (0%)
**Tsimane** reference HAZ						
(0–5)	43	0.18	0.52	(-1.83–0.97)	0 (0%)	0 (0%)
(6–11)	27	0.17	0.55	(-0.70–1.05)	0 (0%)	0 (0%)
(12–23)	60	0.23	0.76	(-1.55–1.69)	0 (0%)	0 (0%)
(24–35)	25	0.06	0.79	(-1.91–1.81)	0 (0%)	0 (0%)
Total:	155	0.18	0.67	(-1.91–1.81)	0 (0%)	0 (0%)
**Tsimane** reference WHZ						
(0–5)	43	-0.44	0.48	(-1.31–1.11)	0 (0%)	0 (0%)
(6–11)	26	0.02	0.57	(-0.90–1.28)	0 (0%)	0 (0%)
(12–23)	60	-0.26	0.69	(-1.88–1.31)	0 (0%)	0 (0%)
(24–35)	25	-0.03	0.86	(-2.28–1.18)	1 (4.0%)	0 (0%)
Total:	154	-0.22	0.67	(-2.28–1.31)	1 (0.6%)	0 (0%)

Tsimane children mean, SD, and prevalence by age group of low (< -2SD) and severely low (< -3SD) WAZ, HAZ, and WHZ scores at initial interviews (n = 156). Z-scores calculated using WHO (2005) reference standards.

For infants aged 0–5 months, rates of low (< -2 SD) and severely low (< -3 SD) WHO WAZ and HAZ ranged from 0.0–2.3%. Between age groups 6–11 and 24–35 months, the total prevalence of moderate to severe underweight (< -2 and < -3 SD WAZ) increased from 12 to 16%, and the total prevalence of moderate to severe stunting (< -2 and < -3 SD HAZ) increased from 15 to 48%. Moderate to severe wasting (< -2 and < -3 SD WHO WHZ) was observed in only five children across all age groups. For Tsimane LMS z-scores, age group was not significantly associated with variance in HAZ (F = 0.40, df = 3, 151, *p* = 0.75) or WAZ (F = 0.77, df = 3, 151, *p* = 0.52), but was associated with variation in WHZ (F = 3.52, df = 3,150, *p =* 0.017). There were no measures of Tsimane HAZ or WAZ below -2 SD (Table 2), and one participant with WHZ < -2.

### Model comparisons

Standardized beta coefficients estimated separately for Tsimane and WHO z-score derived models generally agreed in the direction, magnitude, and significance of many, but not all independent variables (see Table 3 for a visual summary of model comparisons, and S1 Table for full model results). WHO and Tsimane-derived HAZ scores were positively and similarly associated in magnitude for maternal height, IBI ≥ 33 vs. < 33 months, parity ≥ 7 vs. 2–6 and CF introduction at 0–3 vs. 4–6 months. In neither WHO or Tsimane-derived models were growth outcomes associated with EBF vs. CF status in children 0–5 months, or village region or birth season in the full mixed-age sample. No additive independent variables examined were associated with WHZ in either WHO or Tsimane models (Table 3).

**Table 3:** Visual summary of associations between outcomes and independent variables in respective WHO/Tsimane LMS models.

Independent Variable	Model	HAZ	WAZ	WHZ
Maternal height	M1 (baseline)	+ / +	+ / +	ns/ns
Infant sex (male)	M1 (baseline)	ns/ns	ns/ns	ns/ns
Infant age (months)	M1 (baseline)	- / ns	- / ns	ns/ns
IBI > = 33 vs. < 33 months (ref)	M2	+ / +	+ / +	ns/ns
First born vs. < 33 months (ref)	M2	+ / ns	ns/ns	ns/ns
7+ vs. 2–6 births (ref)	M3	+ / +	+ / +	ns/ns
Primiparous vs. 2–6 births (ref)	M3	+ / ns	ns/ns	ns/ns
# siblings < 5 years old	M4	- / ns	- / ns	ns/ns
Remote vs. near market village (ref)	M5	ns/ns	ns/ns	ns/ns
Rainy vs. dry birth season (ref)	M6	ns/ns	ns/ns	ns/ns
CF vs. EBF	M7	ns/ns	ns/ns	ns/ns
CF 0–3 vs. 4–5 months	M8	+ / +	ns / +	ns/ns
Weaned vs. breastfeeding	M9	ns / -	ns/ns	ns/ns

Chart symbols: “+” = significant or trending positive association (p < 0.10), “-” = significant negative association, “ns” = non-significant. Red square = disagreement in significance of association in WHO/Tsimane LMS models; green square = agreement in significance. Models M2-M9 show association of additive independent variable only (all controlled for maternal height, infant sex and age).

**Fig 2:**
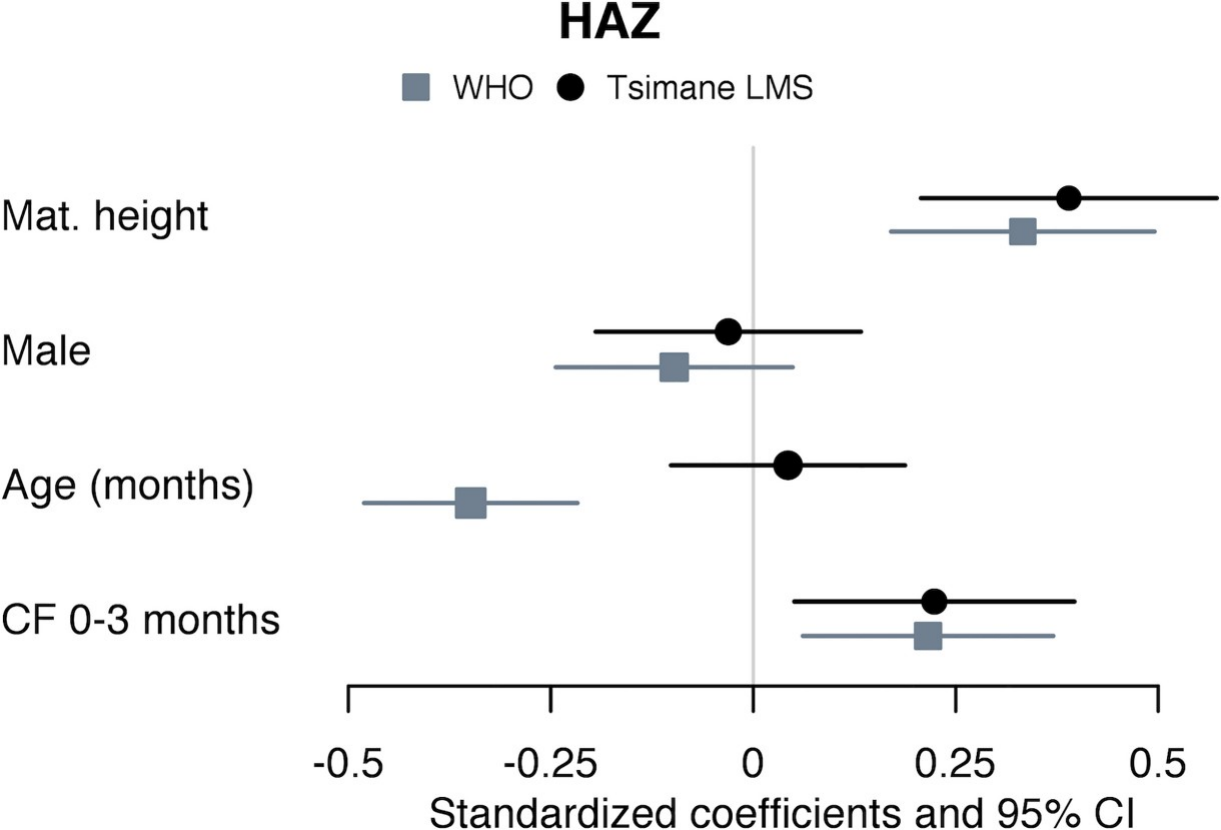
Forest plot of coefficient estimates from Models 8a-8b (mean and 95% CI). Mixed-effects liner regressions on WHO- and Tsimane-derived HAZ scores in children 6–35 months, with random intercept for subject (n subjects = 129; n observations = 198). Independent variables included maternal height (cm); infant sex (male vs. female); child age at measurement; and age at complementary feeding introduction (CF 0–3 months vs. CF 4–5 months).

**Fig 3:**
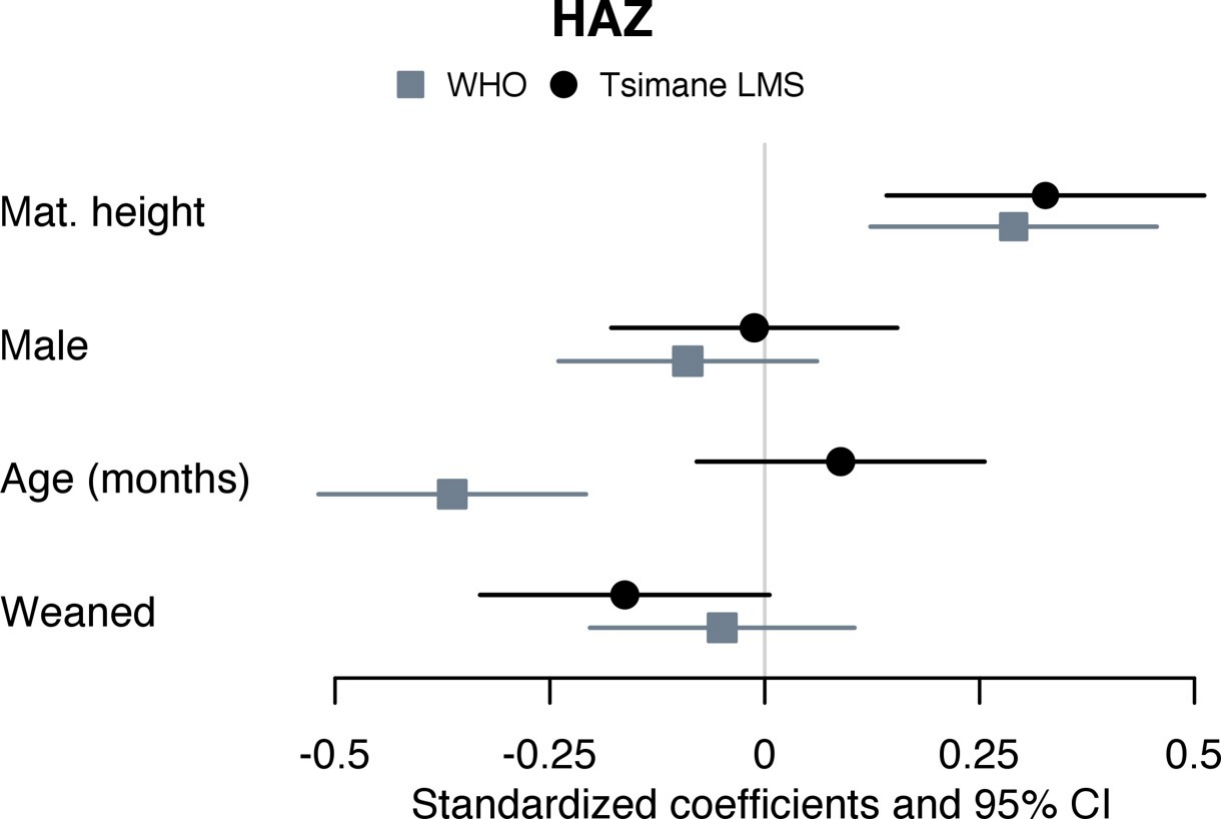
Forest plot of coefficient estimates from Models 9a-9b (mean and 95% CI). Mixed-effects liner regressions on WHO- and Tsimane-derived HAZ scores in children 6–35 months, with random intercept for subject (n subjects = 129; n observations = 198). Independent variables included maternal height (cm); infant sex (male vs. female); child age at measurement; and weaning status (weaned vs. still breastfeeding).

WHO-derived outcomes differed in specific models estimating significant associations with sex (HAZ and WAZ Models 2–3 in S1 Table), primiparous vs. 2–6 births (HAZ Model 3 in S1 Table), number of siblings under age 5 (HAZ and WAZ Model 4 in S1 Table), CF at 0–3 vs. 4–5 months (WAZ Model 8 in S1 Table), and weaned vs. breastfed (HAZ Model 9 in S1 Table). Child age was strongly and negatively associated with WHO but not Tsimane z-scores in all HAZ and WAZ models run on the full, mixed-age sample (Models 1–6 in S1 Table), and with HAZ in models restricted to children ages 6–35 months (Models 8a-b, 9ab in S1 Table). Age was positively associated with WHO and Tsimane-derived HAZ, WAZ, and WHZ among infants 0–5 months, though the association was significant only in Tsimane models. Fig 2 illustrates similarities in Model 8a-b (S1 Table) for HAZ in association with all coefficients except for age. Fig 3 illustrates differences estimated in Model 9a-b (S1 Table) for HAZ in association with age and weaning status

**Fig 4:**
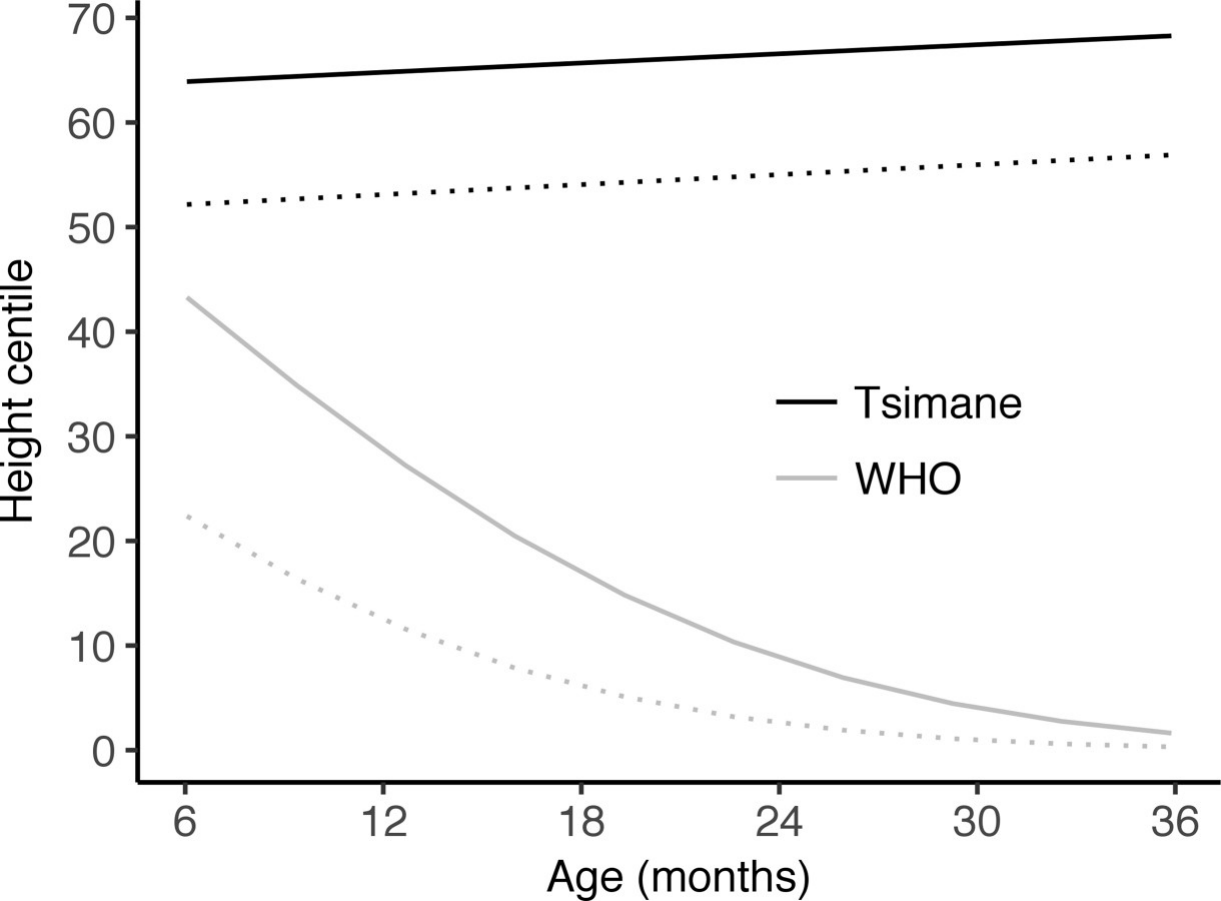
Predicted WHO- and Tsimane LMS-derived height centiles by age and CF status. Predicted values derived from Models 8a-b. Black lines = centiles predicted from Tsimane LMS curves; grey lines = centiles predicted from WHO LMS curves (for both sets, solid = introduced CF at 0–3 months, dashed = introduced CF at 4–5 months).

Removing age from Model 2a but retaining maternal height, infant sex, and IBI did not change the effect of IBI, but resulted in a much poorer model fit: AIC increased from 788 to 844, while the marginal R^2^ decreased from 33% to 10% (Age interactions in S1 Table). Conversely, removing age from the corresponding Tsimane model resulted in a slightly improved model fit, with no change in the estimated effect of IBI (Age interactions in S1 Table). Similar results were obtained by removing age from models of the effect of age of CF introduction on HAZ (Age interactions in S1 Table). Although interaction terms for age and CF were not significant, WHO and Tsimane models predicted different effects of CF on HAZ across infant ages (Fig 4). In Tsimane LMS models, children introduced CF earlier are predicted to have consistently higher HAZ scores across ages. At 9, 26, and 36 months, predicted height centiles for female children with mothers of average height and CF at 0–3 vs. 4–6 months were: 64^th^, 67^th^, 68^th^ vs. 52^nd^, 55^th^, 57^th^.

In contrast, WHO models predicted declining effects of CF on HAZ with age. At 9 and 26 months and holding other model terms constant, heights of children introduced CF at 0–3 vs. 4–6 were predicted at the 35^th^ and 7^th^ vs. the 16^th^ and 2^nd^ centiles. By 30 months, predicted height centiles converge to zero for both CF groups. Weaning, which was negatively associated with Tsimane-derived HAZ, was associated with WHO HAZ only after removing age from the model (Age interactions in S1 Table). There was a significant interaction between age and weaning status on WHO-derived HAZ (Age interactions in S1 Table), but component terms were highly correlated (variance inflation factors > 20).

Largely owing to differences in predicted age and sex effects, marginal R^2^ values (total model variance explained by fixed effects) were consistently larger for WHO as compared to Tsimane LMS models. In other words, because the WHO references produced more variance in z-scores, and this variance was due to divergence from the references with sex and age, these two parameters necessarily accounted for a large proportion of the variance when added to the models. For example, fixed effects included in the baseline model (Models 1a-f, which include maternal height, infant sex, and infant age) explained 27%, 15%, and 1% of total variance in WHO-derived HAZ, WAZ, and WHZ scores, but 10%, 6%, and 1% of variance in corresponding Tsimane LMS scores (S1 Table). After adding IBI (Models 2a-f in S1 Table), marginal R^2^ values increased to 33%, 20%, and 2% in, respectively, WHO HAZ, WAZ, and WHZ models, and to 15%, 10%, and 2% in corresponding Tsimane models.

## Discussion

Accurate identification of local factors associated with child growth variance is relevant to anthropological research on child health disparities and fitness attributes [20], as well as for the development of population-specific interventions to improve child health [26].We examined if the use of WHO standards vs. robust within-population references differently influences growth determinant estimates in a mixed-age sample of indigenous Tsimane children. The standardized coefficients estimated by WHO and Tsimane models agreed across many models, however specific models differed in estimates of total variance explained, as well as the magnitude and significance of some coefficients (Table 3, S1 Table).

Particularly for HAZ, differences in model outcomes largely reflect greater age-related variance in WHO as compared to Tsimane-derived z-scores. These results are not wholly unexpected given the respective reference populations [4], and the fact that Tsimane LMS scores are pre-adjusted for within-population age and sex specific growth. In previous side-by-side comparisons, Tsimane mean 50^th^ centile values for height were equivalent to WHO 10^th^ centiles up through age two. Tsimane and WHO height velocity curves were similar until about three months of age, but then fall between WHO 10^th^ and 35^th^ percentiles up through age two [12]. Similarly, in this study, WHO and Tsimane HAZ scores did not substantively vary before 6 months of age (Models 7a-b in S1 Table), but age was consistently and negatively associated with WHO HAZ in mixed-age samples (Models 1–6 and 8-9a-b in S1 Table). The patterns observed here and previously may reflect the growth-inhibiting effects of infectious diseases and associated inflammatory responses generally during critical growth periods in infancy and early childhood [49–51].

Age-related increases in growth faltering relative to the WHO standards have been widely observed across low- and middle-income populations, reflecting systemically poorer nutritional and pathogenic conditions that critically influence growth prenatally and during the first 1000 days of life [21–24]. Knowing this, researchers may control for age or age group in regression models investigating growth determinants in mixed-age samples [28,29]. However, our results suggest additional methodological approaches may be warranted. For example, Tsimane children who are weaned are smaller than those who are still breastfeeding, though this relationship is only apparent in Tsimane LMS models. The main factors influencing weaning likelihood among the Tsimane are advancing child age and subsequent maternal pregnancy—in itself a factor influenced by the time elapsed since birth [47,52]. Removing age from the Tsimane model weakens the association between HAZ and weaning but slightly improves model fit, while removing weaning does not affect the age term (Age interactions in S1 Table). In contrast, adjusting for age confounds the relationship between weaning and HAZ in the WHO models, but removing age results in a substantially poorer model fit (Age interactions in S1 Table, Fig A in S1 Fig). Such a difference could influence modeling decisions or inferences in studies in which age-related confounding was not specifically being considered.

Age and age-related variables may be irrevocably confounded in mixed-age samples, or require larger sample sizes, more complex non-linear age terms, or more discrete variable measures to assess interactions or lagged effects. We also suggest that while greater variability generally reduces statistical power, associations for some variables—as appears to be the case for siblings under the age of five—may be disproportionately influenced by more extreme measures resulting from negative skew in WHO HAZ distributions (Fig B in S1 Fig). Studies with small sample sizes may be particularly susceptible to biasing due to extreme measures and age-confounding.

The two variables most robustly associated with growth outcomes in both WHO and Tsimane models—relatively longer IBIs and relatively earlier CF—merit additional brief commentary. The association between shorter prior IBI and lower HAZ is consistent with previous research linking shorter prior IBI to poorer child nutritional status and greater morbidity and mortality risks [40,53]. Future research in this population should consider if and how IBI correlates with risks at specific stages-—e.g. reflecting a relationship between restricted fetal growth and size in early infancy, or sibling competition affecting growth at later stages.

We had hypothesized that early CF would be associated with poorer growth outcomes, due to likely increased pathogen exposure and reduced protective immunity and nutritional buffering from breastmilk [54–59]. However, CF relative to EBF was not associated with any growth outcomes in Tsimane infants aged 0–5 months, while earlier CF introduction was associated with greater mean HAZ in children 6–35 months of age (Model 8a-b in S1 Table). These results were robust to both WHO and Tsimane LMS models, suggesting that systemic growth faltering after 6 months in this population should not be attributed to the transition from EBF to CF. The relationship between earlier CF introduction and better growth outcomes may reflect reverse causality as has been observed elsewhere—i.e. with faster growing infants introduced complementary feeding earlier [60–63]. Continued intensive breastfeeding among the Tsimane may be sufficient to buffer additional infectious risks, or the quantity of foods and liquids given may be negligible enough to supplement without supplanting breast milk intake [47]. Additional longitudinal measures of infant size before and after CF introduction are needed to better assess the relative costs and benefits of early CF in this population.

These and other model associations should be cautiously interpreted, however, as our main objective was to assess differences in model estimates using WHO standards vs. Tsimane LMS references. We did not consider different modeling methods [4,64], adjust for multiple comparisons [65], or perform any variable selection procedures to determine the best approximating models [66]. We also stress that the differences in coefficients and significance estimates between WHO vs. Tsimane models were generally modest, and may be further minimized by a larger sample size or adjusted p-values. Our model comparisons must be reproduced in other populations to corroborate our conclusions regarding statistical inferences.

If our results are substantiated, the methodological implications may be particularly relevant for researchers working in small-scale populations and interested in fitness-relevant or modifiable environmental factors that influence growth outcomes. We have observed that variance in Tsimane children’s WHO HAZ scores, which ultimately reflect differences in growth relative to an optimal potential, does not scale linearly with variance in stature in their own community. Population-wide genetic and environmental factors that influence systemic deviation from WHO standards may have little bearing on within-population variation in biological fitness or health outcomes [20]. As a result, variation in factors associated with differences in WHO-derived but *not* within-population-derived growth outcomes may have little biological relevance locally. As another example, WHO WAZ was lower in Tsimane males, which could be interpreted as reflecting sex-biased parental investment. However, growth curves for male and female Tsimane children do not substantially differ before 5 years of age [12], and sex differences were not apparent in Tsimane models. Tsimane boys may deviate from WHO standards to a greater extent than do girls for a variety of reasons, but sex-biasing in local behavioral or other growth determinants would only be inferred if sex had been a predictive factor of Tsimane WAZ scores.

If the research objective is to assess local determinants of variability in growth outcomes, longitudinal changes in age-specific cohorts or within-population growth references should ideally be used. Of course, adequately powered longitudinal studies are often not logistically feasible, and robust, up-to-date references are not available for many populations. Hermanussen and colleagues have devised a method for generating synthetic LMS growth reference charts for any population using limited sets of mean measurements [67]. Another approach might be to integrate the modelling of local growth curves and predictors of growth into a single model. This should ideally be more sophisticated that simply modelling, for example, height as a dependent variable with a linear age term in the model, since growth is rarely linear; rather nonlinear fits (i.e. using GAMLSS) should be used. Bayesian approaches (i.e. STAN/brms, MCMCglmm) might also be useful for comparing or model averaging the posterior parameter estimates obtained with different growth modelling procedures.

Additional methods can also be employed to better estimate and substantiate the biological relevance of WHO-derived growth outcomes. For example, WHO HAZ scores and their determinants may be underestimated in statistical models run on mixed age samples (0–59 months), as the standard deviations used to derive them increase with age [25] and infants aged 0–23 months have been only partially exposed to harmful or protective environmental factors that cumulatively influence growth [26]. These biases may be avoided by analyzing absolute height-for-age differences (i.e. difference between observed height and WHO reference value) [25] and running separate multiple regressions for children 0–23 and 24–59 months of age [26]. In our study, WHO WHZ scores were approximately normally distributed and produced more concurrent results between WHO and Tsimane models. WHZ may be a less biased proxy of within-population variability in size, though there we observed very little variability in WHZ in our study. Finally, researchers can emphasize the biological relevance, rather than statistical significance of, observed variation in WHO z-scores by *a priori* establishing clinically or epidemiologically relevant thresholds for interpreting coefficient estimates of WHO- or within-population-derived z-scores [20].

In closing, we stress that the WHO growth standards remain ideal for between-population comparisons and assessment of large-scale health interventions or secular trends that may impact the prevalence of poor or excess nutrition within specific populations. We do not dispute that Tsimane children’s smaller sizes relative to those of children living under more optimal conditions are in large part due to modifiable environmental conditions that severely impact their health [68,69]. However, these conditions are experienced similarly across ages and families. Individual, maternal, and household factors likely exert more influence on local variation in growth outcomes than these systemic conditions. Age-related skew in WHO HAZ scores may hinder accurate identification of these factors and the magnitude of their effects. For future studies with this population, Tsimane LMS z-scores will likely be more accurate than WHO z-scores in identifying local growth determinants and individuals with locally aberrant growth patterns [12]. Researchers working with other small-scale populations may consider other approximate growth references or additional methodological steps when examining the influence of local growth determinants.

## Supporting information

S1 Text Independent variable selection & category construction. (DOCX)

S1 Table Summary results for Models 1a-9f & age interactions. Full results for all regression models and age interactions in select models.
(XLSX)

S1 Fig Supplementary Figures. Scatter plots of WHO and Tsimane-derived HAZ scores showing differences in age interactions with CF and number of siblings.
(PDF)
