# Supplementary material for: Differences in Tsimane children’s growth outcomes and associated determinants as estimated by WHO standards vs. within-population references
Source: PLoS One. 2019 Apr 17;14(4):e0214965. doi: 10.1371/journal.pone.0214965 (PMC6469771; doi:10.1371/journal.pone.0214965)
Supplement: S1 Text — (DOCX) [file pone.0214965.s001.docx]

**Supplementary text: Independent variable selection & category construction**

All models included child sex, age, and maternal height, which is strongly, positively correlated with offspring nutritional status owing to shared genetic and environmental conditions (Frojo et al. 2014).

**Maternal, infant, and ecological variables examined in models including all participants ages 0-35 months (Models 1-6a-e):**

Few Tsimane women use contraceptives and the average total fertility rate is 9.1 births; high fertility is also valued as a source of agricultural labor and a marker of high status (McAllister et al., 2012). The average age at first birth is 18 years and the mean interbirth interval (IBI) is 30 months (following previous child surviving to one year) (McAllister et al., *in prep*). Short birth to pregnancy intervals, primiparity and high parity (varyingly defined in the literature as five, seven or eight or more births) have been associated with greater risk of adverse offspring outcomes, including undernutrition (Aliyu et al., 2005; Haaga 1989; WHO 2007). To examine the effect of the time elapse between the previous and current birth on an ego child’s growth outcomes, we created a preceding interbirth interval (IBI) category in accordance with WHO (2007) recommendations for minimum birth to pregnancy intervals: (a) first born; (b) IBI less than 33 months (reference group); (c) IBI 33 months or longer. To evaluate expected non-linear associations between parity and child nutritional status, we created a nominal birth order category with three levels: first born, low (2-6 births), and high (7+ births). These levels also correspond to sample interquartile ranges and approximate parity ranges associated with EBF durations in the same sample in previous research (Martin et al., 2016).

In addition, Tsimane infants born in the rainy season have higher morbidity risks than those born in the dry season (Brabec et al, 2018; Gurven 2012), suggesting seasonal nutritional or pathogenic conditions may negatively impact gestational or postnatal growth. Births from May- October of any year were categorized as “dry season” births and those from November - April as “rainy season” births, based on previously observed monthly rainfalls. Average monthly rainfalls for the dry and rainy seasons from 2009 – 2012 were 63.8 and 223.7 cm, respectively (<http://www.senamhi.gob.bo/sismet/>). More remote Tsimane villages have poorer dietary diversity, reduced medical care access, and higher morbidity and mortality (Gurven et al., 2007, 2017; Gurven 2012). Village region was categorized as “near town” if a village was within 20 km from San Borja and accessible by boat or auto (1-3 hours travel), and “remote” if the village was more than 30 km from San Borja, and only accessible via boat or on foot (6-12 hours travel). Finally, while by age seven Tsimane children contribute substantially to household childcare (Winking et al., 2009) a greater number of dependents under age five may more severely constrain maternal or household resources and increase pathogen risk from siblings, resulting in poorer growth outcomes.

**Infant feeding practices examined in models with age-specific subsamples (Models 7-9a-e):**

Exclusive breastfeeding (EBF) to six months and breastfeeding for two years or more may protect infants from infectious and nutritional morbidity (WHO, 2008). Tsimane mothers practice intensive, prolonged breastfeeding with gradual weaning ^9^, but relatively early introduction of complementary feeding (CF) may increase pathogen exposure through poor hygiene and the absence of refrigeration and clean water sources. Full weaning may compound nutritional deficits at any age owing to relatively low diversity of complementary foods (Martin, 2015).

EBF duration was analyzed in two different models, using current breastfeeding status (EBF vs. CF) for infants 0-6 months of age (Models 7a-2), and age of CF introduction at 0-3 vs. 4-6 months for children aged 6-35 months (Models 8a-e). The latter dichotomy reflects the distribution of relatively earlier vs. later ages CF in this population (mean age of CF at 4.4 months), with mothers reporting CF at 0-3 months also more likely to report perceived low milk supply and poor infant growth (Martin et al., 2016). Weaning status (BF vs. weaned) was also evaluated in association with nutritional status in children 6-35 months (Models 9a-e), as no infants were weaned before six months of age (Martin et al., 2016).

**References Cited**

Aliyu, M.H., Jolly, P.E., Ehiri, J.E., Salihu, H.M., 2005. High parity and adverse birth outcomes: exploring the

maze. Birth 32, 45–59. doi:10.1111/j.0730-7659.2005.00344.x

Brabec M, Behrman JR, Emmett SD, Gibson E, Kidd C, Leonard W, Penny ME, Piantadosi ST, Sharma A,

Tanner S, Undurraga EA, Godoy RA. 2018. Birth seasons and heights among girls and boys below 12 years of age: lasting effects and catch-up growth among native Amazonians in Bolivia, Annals of Human Biology, 45:4, 299-313.

Frojo G.A., Rogers, N.G., Mazarieg, M., Keenan, J., Jolly, P. Relationship between the nutritional status of

breastfeeding Mayan mothers and their infants in Guatemala. Mat Child Nutr 10:245-252.

Gurven, M., Kaplan, H., Supa, A.Z., 2007. Mortality experience of Tsimane amerindians of Bolivia: Regional

variation and temporal trends. Am. J. Hum. Biol. 19, 376–398.

Gurven, M., 2012. Infant and fetal mortality among a high fertility and mortality population in the Bolivian

Amazon. Soc. Sci. Med. 75, 2493–2502.

Gurven M, Kaplan H, Stieglitz J, Trumble B, Blackwell AD, Beheim B, Hooper P. 2017. The Tsimane Health

and Life History Project: Integrating anthropology and biomedicine. Evol Anthropol 26:54–73.

Haaga, J.G., 1989. Mechanisms for the Association of Maternal Age , Parity , and Birth Spacing with Infant

Health, in: Parnell, A. (Ed.), Contraceptive Use and Controlled Fertility: Health Issues for Women and

Children Background Papers. National Academies Press, Washington DC, pp. 1–39.

Martin, MA. 2015. Optimal exclusive breastfeeding duration: Evidence of conflict and congruence in tsimane

mother-infant pairs. Ph.D., University of California, Santa Barbara, 2015, 341; 10011385

Martin MA, Garcia G, Kaplan HS, Gurven MD. 2016. Conflict or congruence? Maternal and infant-centric

factors associated with shorter exclusive breastfeeding durations among the Tsimane. Soc Sci Med 170.

Winking, J., Gurven, M., Kaplan, H., Stieglitz, J., 2009. The goals of direct paternal care among a South

Amerindian population. Am. J. Phys. Anthropol. 139, 295–304.

World Health Organization, 2007. Report of a WHO technical consultation on birth spacing: Geneva,

Switzerland 13-15 June 2005 (No. WHO/RHR/07.1). Geneva: World Health Organization.

World Health Organization (WHO), 2008. Indicators for assessing infant and young child feeding practices:

conclusions of a consensus meeting held 6-8 November 2007 in Washington DC, USA. World Health Organization (WHO).
