# Supplementary material for: Differences in Tsimane children’s growth outcomes and associated determinants as estimated by WHO standards vs. within-population references
Source: PLoS One. 2019 Apr 17;14(4):e0214965. doi: 10.1371/journal.pone.0214965 (PMC6469771; doi:10.1371/journal.pone.0214965)
Supplement: S1 Fig — Scatter plots of WHO and Tsimane-derived HAZ scores showing differences in age interactions with CF and number of siblings. (PDF) [file pone.0214965.s003.pdf]

S3 Figure A. Scatter plots of WHO and Tsimane HAZ scores by age and weaning status. Y-axis = height-for-age z score; x-axis = child age (months). Red = still breastfeeding; blue = weaned.

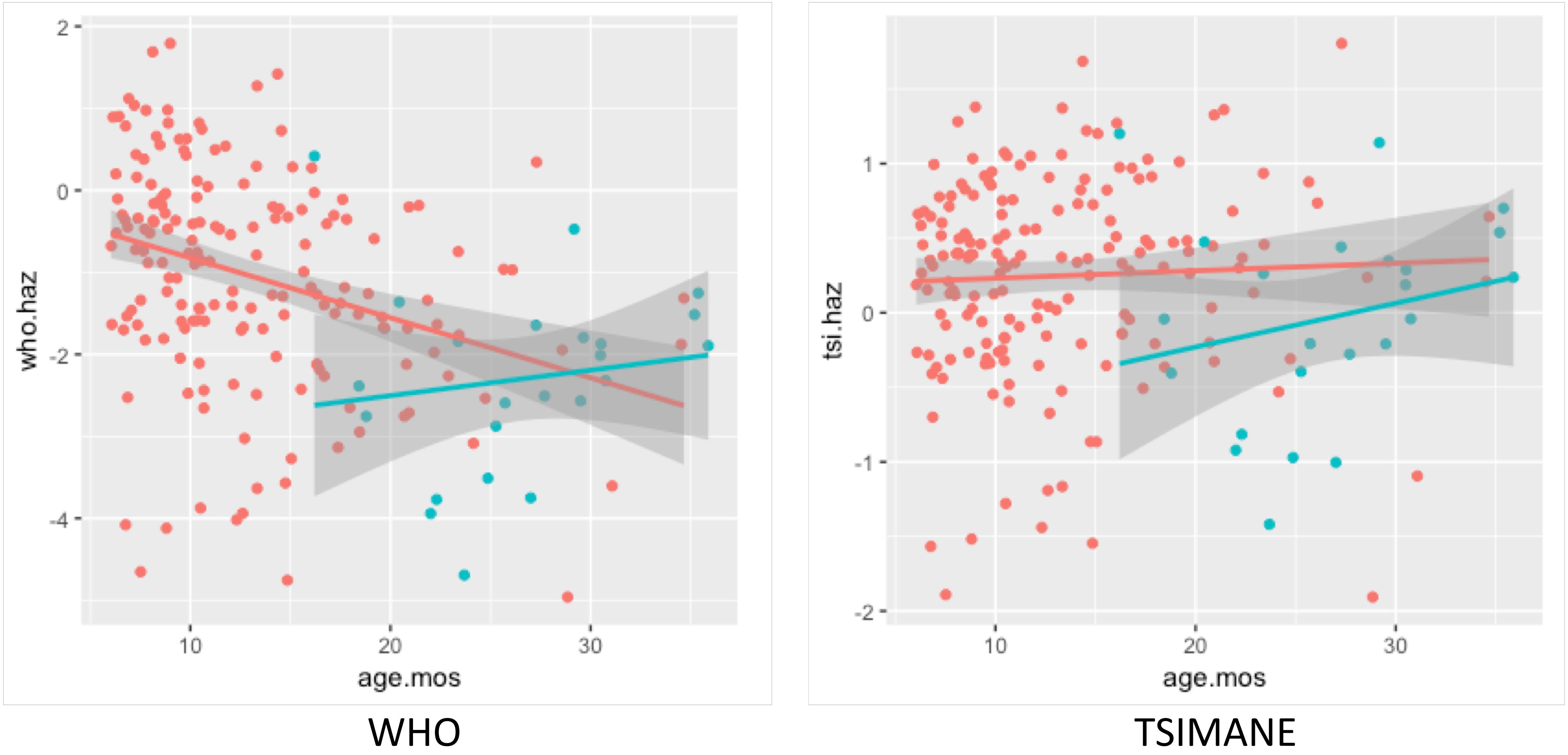

S3 Figure B. Scatter plots of WHO and Tsimane HAZ scores by age and number of siblings age 5 or younger. Y-axis = height-for-age z score; x-axis = child age (months). Red = 0 siblings <= age 5, green = 1 siblings, blue = 2 siblings, purple = 3 siblings

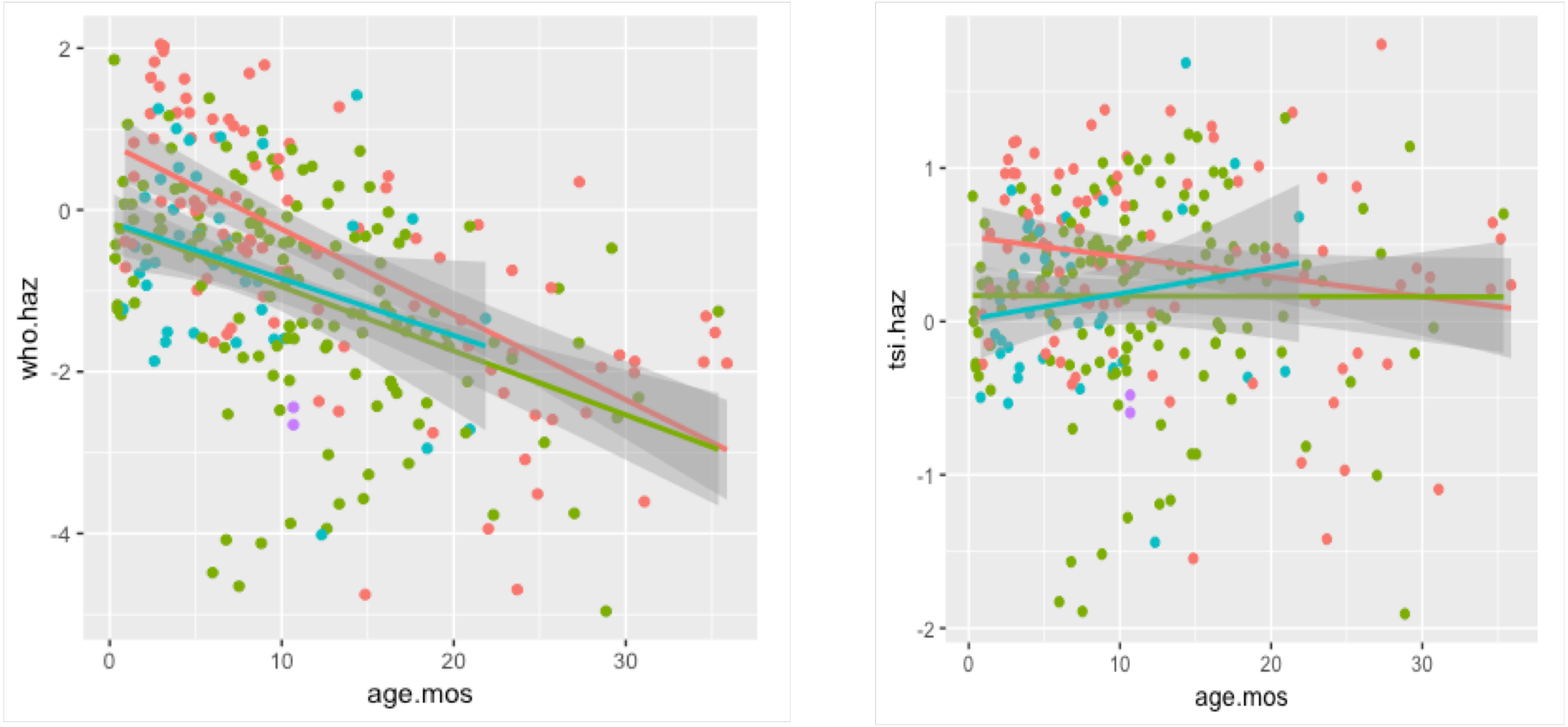

WHO

TSIMANE
